# Urbanization-Related Environmental Factors and Hemorrhagic Fever with Renal Syndrome: A Review Based on Studies Taken in China

**DOI:** 10.3390/ijerph20043328

**Published:** 2023-02-14

**Authors:** Shujuan Li, Lingli Zhu, Lidan Zhang, Guoyan Zhang, Hongyan Ren, Liang Lu

**Affiliations:** 1 National Institute for Nutrition and Health, Chinese Center for Disease Control and Prevention, Beijing 100050, China; 2 State Key Laboratory of Resources and Environmental Information System, Institute of Geographic Sciences and Natural Resources Research, Chinese Academy of Sciences, Beijing 100101, China; 3 Department of Public Health, Faculty of Medicine, Imperial College London, London W2 1PG, UK; 4 Beijing Dong Cheng Center for Disease Control and Prevention, Beijing 100010, China; 5 State Key Laboratory of Infectious Disease Prevention and Control, National Institute for Communicable Disease Control and Prevention, Chinese Center for Disease Control and Prevention, Beijing 102206, China

**Keywords:** HFRS epidemic, urbanization, environmental influencing factors

## Abstract

Hemorrhagic fever with renal syndrome (HFRS) is a rodent-borne disease that has threatened Chinese residents for nearly a century. Although comprehensive prevent and control measures were taken, the HFRS epidemic in China presents a rebounding trend in some areas. Urbanization is considered as an important influencing factor for the HFRS epidemic in recent years; however, the relevant research has not been systematically summarized. This review aims to summarize urbanization-related environmental factors and the HFRS epidemic in China and provide an overview of research perspectives. The literature review was conducted following the PRISMA protocol. Journal articles on the HFRS epidemic in both English and Chinese published before 30 June 2022 were identified from PubMed, Web of Science, and Chinese National Knowledge Infrastructure (CNKI). Inclusion criteria were defined as studies providing information on urbanization-related environmental factors and the HFRS epidemic. A total of 38 studies were included in the review. Changes brought by urbanization on population, economic development, land use, and vaccination program were found to be significantly correlated with the HFRS epidemic. By changing the ecological niche of humans—affecting the rodent population, its virus-carrying rate, and the contact opportunity and susceptibility of populations—urbanization poses a biphasic effect on the HFRS epidemic. Future studies require systematic research framework, comprehensive data sources, and effective methods and models.

## 1. Introduction

Entering the 20th century, climate change, globalization, and urbanization greatly affect the occurrence and development of infectious diseases. The newly emerging infectious diseases (SARS [1], MERS [2], COVID-19 [3,4], etc.) and the resurgent infectious diseases (plague [5], etc.) exert unprecedented impact on the world, enormously challenging human health, social stability, and economic development [6]. Hemorrhagic fever with renal syndrome (HFRS) is a rodent-borne infectious disease caused by different serotypes of Hantaviruses, characterized by fever, hemorrhagic tendency, and kidney damage [7]. China is the country most seriously threatened by the HFRS epidemic in the world (113,961 cases were reported from 2011 to 2020, and the average incidence rate was 0.831/100,000 [8]), accounting for 90% of the total cases worldwide [9], with cases reported in most provinces throughout the country, even in Hong Kong [10] and Taiwan [11].

China has experienced different HFRS epidemic stages during the urbanization process [12]. There were two large-scale HFRS outbreaks in the 1950s and 1980s [13]. Entering the 21st century, the annually HFRS incidence decreased significantly [14], reaching the lowest level in 2009 (0.66/100,000) [15]; however, since then, HFRS incidence has gradually increased (0.71–0.99/100,000) [15]. Although comprehensive prevent and control strategies including vaccination and rodent control measures were enacted, HFRS incidence still fluctuated at a high level [8]. In 2021, the HFRS epidemic (2657 cases and 14 deaths) in Xi’an City attracted wide public attention during the COVID-19 pandemic [16]. The HFRS epidemic remains a serious threat to public health in China.

As a zoonotic disease, the HFRS epidemic is greatly affected by the interaction between humans, the environment, and hosts [5]. In previous studies, natural environmental factors, including climate [14], meteorological factors [17,18,19], landscape [20,21] and land use [22,23,24,25,26,27], and vegetation [28,29], and socioeconomic factors, including human population [30,31], economic development [25,32,33], medical and health conditions [26], and vaccination [34,35], were discussed as influencing factors of the HFRS epidemic. However, there has been limited research on the relationship between urbanization and the HFRS epidemic, while the urbanization process in China has been advancing at an unprecedented speed. Since the Reform and Opening up Policy was implemented in 1978, the proportion of the population living in cities and towns rose from 17.90% in 1978 [36] to 63.89% in 2020 [37], and over 118,205 km^2^ of croplands were converted into impervious surfaces between 1978 and 2017 [38]. Urbanization brings increasing rural–urban migration, population aggregation, and land use change, which deepens the rodent–human–environment interaction and increases HFRS infection [25,39]. At the same time, urbanization brings long-term improvement in sanitary conditions and medical care, which may reduce the HFRS infection rate and incidence of severe cases [30].

Here, we summarize urbanization-related environmental factors and the HFRS epidemic in China in an attempt to improve our understanding of urbanization and the HFRS epidemic and make suggestions for targeted HFRS prevention and control strategies.

## 2. Materials and Methods

### 2.1. Study Selection

We searched the MEDLINE online database (via PubMed, *n* = 415), Web of Science (*n* = 155), and China National Knowledge Infrastructure (CNKI, *n* = 1028 for Preventive Medicine and Hygienics subclass) for articles with the key words “hemorrhagic fever with renal syndrome” or “HFRS” and “China” that were published before 30 June 2022.

### 2.2. Inclusion and Exclusion Criteria

Inclusion criteria were defined as studies providing information on urbanization-related environmental factors and the HFRS epidemic. The inclusion criteria were (i) publications in both English and Chinese; (ii) study location in China; (iii) providing information on urbanization-related environmental factors and the HFRS epidemic; (iv) the effect directions of urbanization-related environmental factors on the HFRS epidemic or HFRS hosts were explicitly stated if applicable; and (v) full text of the article can be accessed.

### 2.3. Data Extraction and Quality Assessment

For each included study, we extracted the following information: the first author, publication year, location (scale), time span, model, related influencing factors, and major results regarding the correlation between urbanization-related environmental factors and the HFRS epidemic. To ensure the reliability of selected publications, two investigators (L. Zhang and L. Zhu) independently screened each publication, and the literature screening process was checked by the principal investigator (S.L.). Inconsistent judgement on a publication was resolved by the principal investigator (S.L.). We adopted a quality assessment form designed based on a similar review [40] to assess the quality of the studies included in this review. The quality assessment table is provided as Supplementary Materials Table S1.

## 3. Results

### 3.1. Overview of Studies Included

A total of 38 articles concerning urbanization-related environmental factors and the HFRS epidemic were included for review. These 38 articles (32 in English and 6 in Chinese) are summarized in Table 1.

### 3.2. The Effects of Urbanization-Related Influencing Factors on HFRS Epidemic

#### 3.2.1. Population Growth and Socioeconomic Development

The most obvious feature of urbanization is population immigration, population increase in urban areas, population density, and urbanization rate growth. Immigrants traveling from non-endemic rural areas might be susceptible to HFRS; at the same time, immigrants newly moving into cities tend to have poor housing and vulnerable medical and health conditions, which increases rodent contact opportunities and HFRS infection risk [30]. A study taken in Hunan Province showed that the number of urban immigrants was strongly positively correlated with HFRS incidence, and the HFRS epidemic in cities with faster economic growth during the urbanization process was prolonged due to the large number of immigrants [30]. A study taken in Hubei Province found that the space gravity center of HFRS cases was consistent with the gravity center of the overall population [31].

Population density was found to be positively correlated with HFRS incidence in Hubei (R = 0.317, *p* < 0.01 [31]; R = 0.372, *p* < 0.01 [45]; R = 0.397, *p* < 0.001 [46]) and Hunan Province (B = 0.437, *p* < 0.01) [43], indicating that the higher the population density, the greater the HFRS epidemic risk. Correlation was also found between HFRS and population density in studies of Shandong Province (contribution rate = 15.9%) [47] and Shaanxi (contribution rate = 8.69%) [25], showing the trend that population density was firstly positively associated with HFRS incidence, then was negatively associated with HFRS incidence when passing the peak, and finally plateaued [25,47]. A study taken in Hunan found a biphasic inverted U-shaped relationship between HFRS and urbanization rate, indicating that HFRS incidence was firstly positively and then negatively correlated with urbanization [30]. A study in Xi’an of Shaanxi Province also found that rapid urbanization greatly affected the HFRS incidence in two different time phases: urbanization firstly increased HFRS infection progressively, but six years later, HFRS infection decreased [26]. The reason for this is that as urbanization develops, it brings improvement in living conditions, development of deratization methods, and an increase in public awareness of rodent prevention and control, which reduce HFRS incidence.

Indicators of socioeconomic development are also remarkable indexes of urbanization. In Hunan Province, GDP was found to be positively associated with HFRS incidence (B = 0.810, *p* < 0.01), which reflected that human urbanization together with social and economic activities increase human–rodent contact risk and also human infection risk [43]. As a principal component, urbanization rate and GDP was found to be negatively associated with HFRS incidence in Chenzhou (R = −0.376, *p* < 0.01) [42]. At the same time, GDP was found to be negatively associated with HFRS incidence in Chongqing (R = −0.0044, *p* < 0.001) [33], Shaanxi (relative contribution rate =5.99%) [25], and Huyi County of Shaanxi (F_1.70,9.02_ =2.917, *p* < 0.05) [32], indicating that as GDP increases, HFRS incidence decreases. Economic development reflected by agricultural mechanization was also negatively associated with the HFRS epidemic (R = −0.383 in Jiaozhou City, *p* < 0.05; R = −0.386 in Jimo City, *p* < 0.05) [44]. A study in Shaanxi Province found that the regression coefficient of the principal component of socioeconomic and population density decreased from positive to negative [41], which also indicated the biphasic effect of urbanization on the HFRS epidemic.

#### 3.2.2. Land Use/Land Cover Change

Land use or land cover change is another prominent feature of urbanization. As people crowd into cities, houses and corresponding infrastructure are built; architectural areas replace cultivated land, grassland, forest land, and other unused land. Intensifying land use change usually occurs at the junction of urban and rural areas. A study in Xi’an proved that the influence of urbanization was normally distributed in the rural–urban fringe areas [26]. On one side, human activity destroys the ecological niche of rodents and brings changes to rodent population density, structure, and habitat; on the other side, abundant human activities in these areas increase human exposure to rodents.

- Artificial facilities construction

Urbanization is accompanied by artificial facility construction including roads, buildings, bridges, and factories. The construction of artificial facilities could change the rodent distribution [48], density [50], and structure [50], which changes the natural HFRS foci and affects human HFRS infection. A study in Guizhou showed that bridge construction gradually diminished the impact of the natural barrier formed by rivers and mountains, with *A. agrarius* in the west bank of the river transitioning from being never detected with the HFRS virus antigen to presenting a high infection rate (13.85%) [48]. Dam construction was also found to have an influence on the HFRS epidemic. A study taken in Chongqing proposed scenarios of the impact of the Three Gorges Dam (TGD) on HFRS transmission as follows: the construction of TGD resulted in significant ecological changes and substantial modifications to the depth and flow pattern of the river, millions of residents were relocated, and local conditions such as the local climate were also affected [33]. The scenarios could not be proven due to data availability. However, another study taken in the TGD region explained that the reason for the lack of an HFRS epidemic outbreak in this area was the effective deratization measures [49]. Construction of an economic development zone could also greatly affect the HFRS epidemic. The construction of Huangdao District as a National Economic and Technological Development Zone greatly changed the HFRS epidemic pattern compared with that of the adjacent Jiaonan County, with the HFRS virus detection rate in Jiaonan (2.81%) significantly higher than in Huangdao (0.00%) (χ^2^ = 172.38, *p* < 0.05) [50]. The comparison of Huangdao and Jiaonan indicated that urbanization changed the ecological conditions of rodents, led to changes in the rodent population, and eventually reduced HFRS incidence.

- Land use change

Land use change driven by anthropogenic activities leads to habitat loss for hosts, which could affect wildlife abundance and HFRS infections [22]. Cultivated land, forest land, grassland, and orchard land provide rodents with natural habitats, especially for wild hosts. At the national level, cultivated land, forest, orchard land, and grassland were positively associated with HFRS incidence [21,56]. Cultivated land was found to be positively associated with HFRS incidence in Beijing (as rice paddies increased by 1%, the HFRS incidence rate increased by 27.8%) [51], Shandong (contribution rate = 9.98%) [47], Hunan (B = 0.435, *p* < 0.01) [43], Hubei (R = 0.421, *p* < 0.01) [31], and Shaanxi (contribution rate = 8.70%) [24]. Risk area in cultivated land was also found in Shandong [54], Shaanxi [25], Changsha [23], Chenzhou [55], Loudi, and Shaoyang City [20] from Hunan. In Hu County of Shaanxi Province, a decreased carrying capacity is associated with loss of farmland area [22].Orchard land was found to be positively associated with HFRS incidence in Beijing (as orchard land increased by 1%, HFRS incidence rate increased by 4.33%) [51]. In the Big Three Gorges of Chongqing, a significant negative correlation was present between land use change in the grass area and the cumulative HFRS incidence (R = −0.676, *p* = 0.011) [57]. In Shandong Province, forest land (contribution rate = 8.71%) and grassland (contribution rate = 11.06%) were found to affect HFRS incidence significantly, with a trend of firstly increased and then decreased incidence [47]. 

Water body presence was found to be positively correlated with HFRS incidence in Hubei [31,45] and Jiangsu [53], while in Shandong, water bodies were found to be firstly positively correlated and then negatively correlated with HFRS incidence [47]. A study in Xi’an found that the association of water bodies with the HFRS incidence rate was higher within the radii of 696.15 m and 1575.39 m [26]. On one side, the water body is beneficial for vegetation growth including crops, which helps to establish thriving rodent populations [31]. On the other side, a water body may play a critical role in changing the humidity and temperature, which further affects the formation and maintenance of the host habitat [26].

Artificial areas and built-up areas provide suitable habitats for rodents, especially for *R. norvegicus*, which is closely related with humans. Artificial area, or built-up land, was found to be positively correlated with HFRS incidence or with higher HFRS risk in Hunan [20,23,43,52,55] and Shaanxi [24,25], where the main rodent type was *R. norvegicus*. At the same time, negative correlations between built-up land and HFRS incidence and between rural settlement and HFRS incidence were found in Beijing [51] and Shandong Province [47]. The negative correlation might be explained by the improvement in sanitary conditions in those residential areas, which could reduce rodent populations and the corresponding infection risk. Artificial areas including highways and railways were also found to be correlated with the HFRS epidemic in Jiangsu Province, which could be explained by the transportation system providing a suitable living environment and ample food for rodents [53].

- Vegetation

Vegetation is an important part of land cover or land use type. Vegetation provides rodents with food and shelter, which affects HFRS incidence via the ecological cascading effect [22]. The ecological cascading effect dictates that an increase in resources would allow the rodent host to survive and reproduce more easily, leading to more infections [22]. With better vegetation, the hosts, especially wild rodents, thrive through access to sufficient food and habitats, which could enlarge rodent populations; areas with less vegetation are usually associated with more human activities, and rodent populations closely related with humans could be easily disturbed in these areas, which might increase human–rodent contact and HFRS infections. Urbanization brings vegetation-related land use change, which could be reflected in vegetation indexes. Normalized difference vegetation index (NDVI) is an indicator of surface vegetation coverage, which represents the comprehensive situation of the natural environment combined with human social and economic activities [43]. NDVI was found to be positively or negatively associated with HFRS incidence. Studies taken in Hunan [29,42,58,59], Inner Mongolia [60], Heilongjiang and Shaanxi Province [58], and in the whole country [56] found positive correlations between NDVI for certain land use types with 1–6 months lag and HFRS incidence or rodent density. Studies taken in Hebei [61] and Hunan Province [23,43] found a negative correlation between NDVI and HFRS incidence. Other studies found that HFRS risk was higher at certain NDVI value sections [21,28,52].

- Livestock husbandry

With a large amount of people living in the city, there is great demand for food and other products, which promotes the development of livestock husbandry. Livestock husbandry farms usually distribute to urban suburbs or developed towns. Husbandry farms provide abundant food and shelter for rodents, which may increase HFRS infections [62]. A study taken in Changchun found that increasing HFRS incidence was significantly associated with livestock husbandry, particularly with deer cultivation (HFRS incidence increased by 70.7% as the deer density increased by 10 head per km^2^ (*p* < 0.001) in Shuangyang County; HFRS incidence increased by 90.4% as the deer density increased by 10 head per km^2^ (*p* < 0.001) in nine combined counties, especially for SEOV-dominated HFRS that occurred from 1998 to 2012 [62]). A study in Shaanxi Province found that pig density was mainly a positive influencing factor on HFRS incidence (contribution rate 9.86%) [25].

#### 3.2.3. Vaccination Program and Rodent Control

The most obvious feature of the improvement in medical and health conditions brought about by urbanization is vaccination. Vaccination programs greatly reduce HFRS infection in targeted people within targeted regions [22]. A study assessed the effects of the implementation of China’s expanded program of immunization (EPI) and found that the proportion of HFRS among individuals targeted by EPI (16–60 years of age) decreased from 86.9% in 2005 to 81.9% in 2010 and the proportion of HFRS cases among the unvaccinated group aged <16 and >60 increased from 13.1% in 2005 to 18.1% in 2010 [34]. The notable characteristic is that in the >60 age group, the proportion rose from 8.8% in 2005 to 14.7% in 2010 [34]. Similar results were found in Yichun of Jiangxi Province [35] and Shaanxi Province [63]. As urbanization attracts a great number of young and middle-aged people to work and live in cities, the children and elderly left behind are not covered by vaccination program effects and are vulnerable to HFRS infection, especially the elderly working in the fields. The study taken in the Three Gorges Reservoir Region demonstrated that rodent population-reducing measures could be effective in controlling rodent-borne diseases during larger-scale hydraulic engineering construction by decreasing rodent density and maintaining it at low levels [49]. 

### 3.3. The Mechanism of Urbanization’s Influence on HFRS Epidemic

Based on the studies reviewed, we summarized the mechanism of urbanization’s influence on the HFRS epidemic. To begin with, “urbanization” refers to the historical process by which a country or region gradually changes from a traditional rural society dominated by agriculture to a modern urban society dominated by nonagricultural industries. The major characteristics of urbanization are population urbanization (increase of total population, population density, and urbanization rate), economic urbanization (GDP growth and other financial index increase), social urbanization (medical and health condition improvement), and environmental urbanization (residential area, industrial areas, road construction, etc.), which were represented as urbanization-related influencing factors of the HFRS epidemic mentioned previously.

As an infectious disease, the source of HFRS infection is host animals infected with Hantaviruses [5,13]. Host animals of transmitted HFRS in China mainly include *Apodemus agrarius*, mainly transmitting the Hantaan virus (HNTV), and *Rattus norvegicus*, mainly transmitting Seoul virus (SEOV) [13], and both rodent species are closely related to human activities. *A. agrarius* mainly inhabits farmland and the forest edges surrounding it, and *R. norvegicus* inhabits human residential areas. Routes of transmission include rodent bites, contact with rodent feces and/or contaminated food, and aerosol transmission [13]. People of both genders and of different ages and occupations are generally susceptible to HFRS, and people working in fields and on construction sites are more likely to become infected [64].

**Figure 1:**
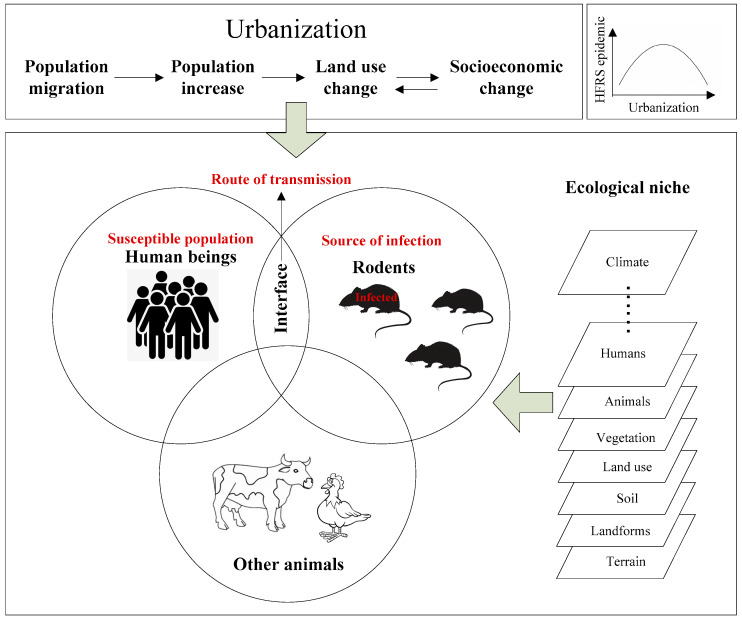
The theoretical model of urbanization influence on the HFRS epidemic.

The HFRS epidemic is affected by urbanization in the following aspects. (i) In the natural environment, human beings and rodents occupy certain ecological niches. The ecological niche refers to a set of physical conditions that permit a positive turnover of the population of an organism; here, we integrated the abiotic and biotic niche definitions [65]. The physical conditions include environmental factors from terrain, landform, soil, land use, vegetation, animals, and humans to meteorological factors and the vertical climate (Figure 1). The ecological niches of human and rodents overlap with each other at certain scales [65]. Urbanization brings increased overlap of the ecological niches of rodents and human beings and particularly affects the ecotones defined as “edges or transitionary zones between adjacent ecological systems where biophysical factors, biological activities and ecological evolutionary processes are concentrated and intensified” [66,67]. This overlapping greatly affects the rodent population [49], its virus-carrying rate [48,50], and contact opportunity [22,34,35,63], and eventually influences the HFRS epidemic. (ii) Urbanization brings improvement in living, medical, and health conditions of humans, and vaccination programs target susceptible people, which combine to greatly reduce the HFRS epidemic by changing the rodent population [50], reducing contact opportunities, and increasing population immunity [34,35]. (iii) Urbanization plays the mentioned roles in a certain order. The early stage of urbanization greatly increases HFRS infection risk, while the mature stage of urbanization decreases HFRS infection risk. It was demonstrated as a “biphasic inverted U-shaped relationship between HFRS incidence and urbanization” in previous studies [26,30].

## 4. Discussion

Nowadays, rapid urbanization is intensifying in developing countries, especially in China. According to the report by the United Nations Development Programme, by 2030, China will add 310 million new urban residents, and the urbanization level will reach 70% [68]. The irresistible trend of urbanization greatly affects the HFRS epidemic by promoting interaction between rodents, humans, and the environment. Evidence proved that urbanizing cities with high economic growth exhibit extended epidemics [30]. With the expansion and deepening of urbanization in China, we could not neglect the influence of urbanization on the HFRS epidemic. However, there were limited studies on the interaction between the HFRS epidemic and urbanization. The effects of urbanization-related environmental factors on the HFRS epidemic had also not been systematically reviewed and summarized. In this review, we summarized urbanization-related environmental factors and the HFRS epidemic in China in an attempt to improve our understanding on urbanization and the HFRS epidemic and make suggestions for future prevention and control strategies and research directions.

Urbanization-related influencing factors were found to have greatly affected the HFRS epidemic in China. As specific indicators of urbanization, population, urbanization rate, GDP, land use change, and comprehensive indicators were used to detect the interaction between the HFRS epidemic and urbanization. In general, socioeconomic status including GDP, population, and complex urbanization index reflected the long-term and overall change in urbanization, while land cover/land use change was the specific change influencing the human–rodent–virus interaction. In the long term, urbanization represented by GDP growth, population increase, and urbanization rate increase played the key role in affecting the HFRS epidemic in China, as indicated by the studies on “urbanization and HFRS epidemic” [26,30]. In studies considering multiple variables (GDP, population, land use, etc.), socioeconomic factors and land use both played important roles, while land use had more direct influence on the HFRS epidemic, with land use (Shaanxi, 38.65% [25]; Wei River Basin, 60.7% [24]; Shandong, 50.94% [47]) or NDVI (Dongting Lake District, 62.7% [52]) contributing to nearly or more than 50% of the effect. As to land use types, cultivated land, grassland, forest land, and orchard land were closely related with wild rodents and HFRS epidemic risk, with the contribution rate varying according to different areas and different scales. Built-up land was found to be the key influencing factor in some area [24,25]. As to land cover indicated by NDVI, the effects on the HFRS epidemic varied among different times, regions, and scales, which provided information for developing accurate prevent and control measures. Vaccination and rodent control were strong control measures to reduce the HFRS epidemic.

By changing the ecological niches of humans—affecting the rodent population, its virus-carrying rate, contact opportunities, and populations’ susceptibility—urbanization greatly influences the HFRS epidemiology. With the social and economic development after the founding of the People’s Republic of China, the HFRS epidemic expanded its scope continuously [69]. The HFRS outbreak in the 1950s and 1980s might be related to large-scale farmland infrastructure construction and agricultural production [13], indicating that human socioeconomic activities and early urbanization greatly impact HFRS and the environment and increase infection. At the same time, some studies showed that in the long run, the urbanization process reduced HFRS infection in China [26,30,50]. The biphasic inverted U-shaped effect of urbanization on the HFRS epidemic indicated that special attention should be paid to vulnerable areas and vulnerable people at certain urbanization stages. In the urbanization expansion stage, urban–rural interfaces are the most vulnerable areas, so rodent control measures and targeted vaccination should be conducted in these areas. At the same time, with young immigrants moving into cities, the elderly left behind undertake more agricultural activities, which increases the HFRS infection risk of this vulnerable population [34]. The typical HFRS epidemic area (Shaanxi, Shandong, Hunan, etc.) where rapid urbanization is taking place should also be given special attention.

As to research directions, previous studies on the relationship between urbanization and the HFRS epidemic were limited to effects of ecological environment change. Interaction between environmental factors, rodents, humans, and viruses should be explored in depth. In addition, the urbanization process could also deeply affect the local climate and indirectly affect the HFRS epidemic [33,70], which should also be explored with specific data. Multivariate data including long time-series remote sensing image data, meteorological monitoring station data, and host surveillance data should be applied in future explorations. Although SEOV is the main Hantavirus in urban areas, there are limited data and research on this topic due to data availability. The direct network reporting system for infectious diseases should include this information to provide data for in-depth study. In addition, rodent surveillance in interfaces should be strengthened and applied in HFRS research.

There were some limitations of this review that should be mentioned. Firstly, in the present review, the included studies were descriptive due to the fact that the factors driving HFRS infection were multifactorial in space and time and autocorrelated, and we usually cannot parse out the effect of one factor without the consideration of another. Further statistical inference and explanatory analysis are needed. Secondly, the HFRS epidemic is also greatly affected by climatic and meteorological factors. The interaction between meteorological, environmental, biological, and anthropogenic dimensions should be further explored. Thirdly, the included studies did not distinguish between HNTV and SEOV types of HFRS due to data limitation. Since urbanization had different influences in different regions by affecting interaction between the environment and specific rodent types, this information would be useful for future research on this topic.

## 5. Conclusions

Urbanization poses a biphasic effect on the HFRS epidemic by promoting interaction between humans, hosts, Hantavirus, and the natural and socioeconomic environment. The effects of urbanization-related influencing factors on HFRS in China varied in different periods and regions according to the specific stage of urbanization. Further exploration on the relationship between urbanization and HFRS prevalence requires more scientific and systematic research framework and ideas, comprehensive data sources, and effective methods and models.

## Figures and Tables

**Table 1:** The effects of urbanization-related environmental factors on the HFRS epidemic in selected studies.

Influencing Factors	Indicators	Area (Scale); Time Span	Model; Influence Effect	Other Factors Considered
Socioeconomic factors	Socioeconomic and population (+, −)	Shaanxi Province (county) [41]; 2005–2018	Geographical temporal weighted regression model; R = 0.07–0.47 (2005), −0.005–0.15 (2011), −0.18–0.19 (2018)	Climate and meteorological factors
	Comprehensive indicator of urbanization level (+, −)	Xi’an City (the total area and county) [26]; 2005–2018	Scatter plots; two characteristic phases, a trend of continuously increasing and then decreasing, the sixth year as the peak	Water body; rainfall; vegetation
	Component representing GDP and the urbanization rate (−)	Chenzhou City (the total area) [42]; 2006–2010	Cross-correlation analysis; R = −0.376, *p* < 0.01	Rodents; NDVI; climate
	Urbanization rate (+, −)	Hunan Province (total province and city level) [30]; 1963–2010	Scatter plot and plotted fitting lines; biphasic inverted U-shaped relationship between HFRS and urbanization	Elevation
Economic	GDP (+)	Hunan Province (1 km grid) [43]; 2010–2019	Binary logistic regression model; B = 0.810, *p* < 0.01 (2nd)	Soil; land use; population (3rd); altitude; NDVI (1st); precipitation
	GDP (−)	Chongqing City (total city) [33]; 1997–2008	Poisson regression models; R = −0.0044, *p* < 0.001	Temperature and rainfall; rodents
		Shaanxi Province (county) [25]; 2005–2016	Multivariate boosted regression; relative contribution 5.99%; negative relationship, with the highest correlation corresponding to low GDPs of 0–2500 Yuan	Meteorological factors; elevation; land use (1st); population density (3rd); pig density (2nd); cattle density; goat density; selenium
		Huyi District (total district) [32]; 1984–2016	Generalized additive models; negative nonlinear correlation between GDP and HFRS incidence (F_1.70,9.02_ =2.917, *p* < 0.05) (1st)	Temperature
	Agricultural mechanization (−)	Qingdao City (total city) [44]; 1985–2015	Pearson correlation; R = −0.383 in Jiaozhou City, *p* < 0.05; R = −0.386 in Jimo City, *p* < 0.05	Rodents
Population	Population density (+)	Hubei Province (county) [45]; 2005–2015	Correlation analysis; R = 0.372, *p* < 0.01	Meteorological factors; rodent density; water area; height
		Hubei Province (county) [46]; 2005–2014	Spearman’s rank correlation analysis; R = 0.397, *p* < 0.001	Meteorological factors
		Hubei Province (county) [31]; 2011–2015	Ordinary least square; R = 0.317, *p* < 0.01	Farmland area; rainfall; water area; average humidity
		Hunan Province (1 km grid) [43]; 2010–2019	Logistic regression model; B = 0.437, *p* < 0.01 (3rd)	Soil; land use; altitude; NDVI (1st); precipitation; GDP (2nd)
	Population density (+, −)	Shandong Province (county) [47]; 2010–2018	Boosted regression tree model; contribution rate = 15.9% (1st), a trend of firstly increasing and then decreasing after the peak	Elevation (2nd); land use (grassland, 3rd); meteorological factors; GDP; NDVI
		Shaanxi Province (county) [25]; 2005–2016√	Multivariate boosted regression; relative contribution = 8.69% (3rd); a trend of firstly increasing and then decreasing after the peak	Meteorological factors; elevation; land use (1st); GDP; pig density (2nd); cattle density; goat density; selenium
	Immigrants (+)	Hunan Province (total province and city level) [30]; 1963–2010	Scatter plot and plotted fitting lines; a positive correlation between HFRS incidence and number of immigrants	Elevation
Land use change—Construction	Bridge construction (+)	Guizhou Province (total area) [48]; 1983–1998	Rat surveillance; HFRS virus antigen infection rate changed from being never detected (0%) to a high level (13.85%)	None
	Dam construction (−)	Three Gorges Reservoir Region (total area) [49]; 1997–2012	Surveillance; annual HFRS incidence rate decreased by 85.74% after the dam impoundment; the indoor rodent density (4.38% to 2.29%, χ^2^ = 193.4, *p* < 0.05) and outdoor rodent density (4.41% to 2.66%, χ^2^ = 188.4, *p* < 0.05) decreased	Rodents
	Economic zone construction (−)	Huangdao and Jiaonan of Qingdao City [50]; 1979–2014	Comparative study (chi-square test); rodent capture rate in Jiaonan (4.00%) vs. Huangdao (0.51%); HFRS virus detection rate in Jiaonan (2.81%) vs. Huangdao (0.00%) (χ^2^ = 172.38, *p* < 0.05)	None
Land use type	Artificial area (building land, urban and rural construction land, highways, railways) (+/−/risk area)	Beijing City (total city) [51]; 1997–2006	Poisson regression; built-up land, negative correlation (−0.82%/1%, as built-up land increased by 1%, the HFRS incidence rate decreased by 0.82%)	Elevation
		Shandong Province (county) [47]; 2010–2018	Boosted regression model; rural settlement contribution = 9.25%, negative correlation	Population density (1st); elevation (2nd); meteorological factors; GDP; NDVI
		Shaanxi Province (county) [25]; 2005–2016√	Multivariate boosted regression; relative contribution = 23.02% (1st); initially increased significantly and then plateaued in response to increase in percentage coverage of artificial area	Meteorological factors; elevation; GDP; land use (1st); population density (3rd); pig density (2nd); cattle density; goat density; selenium
		Wei River Basin of Shaanxi Province (5 km grid) [24]; 2005–2015√	Boosted regression model; building land contribution = 49.10% (1st), positive correlation	Elevation (2nd); farmland
		Dongting Lake District (nearly 1 km) [52]; 2005–2010	Ecological niche models; cumulative contribution rate of land use = 7.9%, construction land was the main risk land type associated with HFRS transmission between June and September	Elevation; meteorological factors; NDVI (1st); land use (2nd); eco-geographical data; human footprint index (3rd); compound topographic index; distance to water source; slope
		Changsha City (nearly 1 km) [23]; 2005–2009	Ecological niche models; risk area in urban land; the risk level is correlated with an increase in area of urban land	Elevation; temperature; precipitation; NDVI; rodent density
		Hunan Province (1 km grid) [43]; 2010–2019	Logistic regression model; B = 0.435, *p* < 0.01, urban and rural construction land as high-risk area for HFRS	GDP (2nd); soil; population (3rd); altitude; NDVI (1st); precipitation
		Jiangsu Province (county) [53]; 2001–2011	Single factor analysis; significant nonlinear correlation between HFRS incidence and distance to railways and highways	Meteorological factors; elevation; NDVI
	Cultivated land (+/risk area)	Wei River Basin of Shaanxi Province (5 km grid) [24]; 2005–2015	Boosted regression model; farmland contribution = 8.70%, positive correlation	Building land (1st); elevation (2nd)
		Beijing City (total city) [51]; 1997–2006	Poisson regression; rice paddies, positive correlation (+27.8%/1%, as rice paddies increased by 1%, the HFRS incidence rate increased by 27.8%)	Elevation
		Shandong Province (county) [47]; 2010–2018	Boosted regression tree model; cultivated land contribution = 9.98%, positive correlation	Population density (1st); elevation (2nd); meteorological factors; GDP; NDVI
		Hu County (total county) [22]; 1984–2014	Bayesian state space approach; decreasing carrying capacity is associated with loss of farmland	Rainfall; resource availability; rodents
		Shandong Province (grid) [54]; 2005–2009	Ecological niche model; contribution rate of land cover = 31.2%, with highest risk in rain-fed croplands and mosaics of vegetation/croplands	Meteorological factors; NDVI; land surface temperature during nighttime
		Shaanxi Province (county) [25]; 2005–2016	Multivariate boosted regression; relative contribution = 13.21%; initially increased significantly and then plateaued	Meteorological factors; elevation; GDP; land use (1st); population density (3rd); pig density (2nd); cattle density; goat density; selenium
		Hunan Province [43]; 2010–2019	Binary logistic regression model; B = 0.435, *p* < 0.01, cultivated land as high-risk area for HFRS	GDP (2nd); population (3rd); soil; altitude; NDVI (1st); precipitation
		Changsha City (nearly 1 km) [23]; 2005–2009	Ecological niche models; risk area in cultivated land; the risk level is correlated with an increase in area of cultivated land	Elevation; temperature; precipitation; NDVI; rodent density
		Loudi City and Shaoyang City (total area) [20]; 2006–2013	Matrix; cultivated land had the largest proportion of cases	Rodents
		Hubei Province (county) [31]; 2011–2015	Ordinary least square; farmland area (R = 0.421, *p* < 0.01)	Population; rainfall; water area; average humidity
		Chenzhou City (total city) [55]; 2006–2015	Matrix; the highest risk of HFRS occurred on cultivated land	Meteorological factors; NDVI; TVDI; rodents
	Forest land (+/risk area)	China (1 km grid) [21]; 1994–1998	Multivariate logistic regression; OR = 2.04, *p* < 0.01	Elevation; NDVI; soil; meteorological factors
		China (province) [56]; 2005–2009	Geographically weighted regression model; positive correlation, *p* < 0.01 in 2006, 2008, and 2009	Meteorological factors; elevation; NDVI
		Shandong Province (county) [47]; 2010–2018	Boosted regression tree model; woodland contribution = 8.71%, initially increased and then decreased	Population density (1st); elevation (2nd); meteorological factors; GDP; NDVI
	Grassland (+/risk area)	Big Three Gorges area (county) [57]; 1997–2007	Spearman correlation; R = −0.676, *p* = 0.011	Rodents
		Shandong Province (county) [47]; 2010–2018	Boosted regression tree model; grassland contribution = 11.06%, initially increased and then decreased	Population density; elevation; woodland; rural settlement; water body
	Orchard land (+/risk area)	China (1 km grid) [21]; 1994–1998	Multivariate logistic regression; OR = 1.97, *p* < 0.01	Elevation; NDVI; soil; meteorological factors
		Beijing City (total city) [51]; 1997–2006	Poisson regression; orchard, positive correlation (+4.33%/1%, as orchard land increased by 1%, the HFRS incidence rate increased by 4.33%)	Elevation
	Water body (+/risk area)	Shandong Province (county) [47]; 2010–2018	Boosted regression tree model; water body contribution rate = 8.63%, initially increased and then decreased	Population density (1st); elevation (2nd); meteorological factors; GDP; NDVI
		Hubei Province (county) [31]; 2011–2015	Ordinary least square; R = 0.087, *p* < 0.01	Population; farmland; meteorological factors
		Jiangsu Province (county) [53]; 2001–2011	Single factor analysis; significant nonlinear correlation between HFRS incidence and distance to rivers and lakes	Meteorological factors; elevation; NDVI
		Xi’an City (the total area) [26]; 2005–2018	Kernel density estimate; higher HFRS incidence rate within the radii of 696.15 m and 1575.39 m	Urbanization; rainfall; vegetation
		Hubei Province (county) [45]; 2005–2014√	Correlation analysis; R = 0.352, *p* < 0.01	Meteorological factors; rodent density; population density; height
Land use—Vegetation	Specific normalized difference vegetation index (NDVI) value as risk area	China (1 km grid) [21]; 1994–1998	Univariate analysis; OR_NDVI = 0.1–0.2_ = 3.69, OR_NDVI = 0.2–0.3_ = 3.99, *p* < 0.001	Elevation; meteorological factors; land surface temperature
		109 counties in China (county and the total area) [28]; 2002.01–2013.12	Quasi-Poisson regression with a distributed lag nonlinear model and multivariate meta-analysis; highest risk in 80th percentile of NDVI	Meteorological factors
		Dongting Lake District (nearly 1 km) [52]; 2005–2010	Ecological niche models; cumulative contribution rate of the monthly average NDVI = 62.7% (1st influencing factor), the highest HFRS incidence was in spring (NDVI between 0.5 and 0.7) and in winter (NDVI between 0.4 and 0.5)	Elevation; meteorological factors; land use (2nd); eco-geographical data; human footprint index (3rd); compound topographic index; distance to water source; slope
	NDVI for specific months or with time lags, or for specific land use types (+)	China (province) [56]; 2005–2012	Geographically weighted regression model; positive correlation, *p* < 0.01 in 2005, 2006, 2007, and 2008	Meteorological factors; elevation; land use
		Chenzhou City (the total area) [42]; 2006–2010	Polynomial distributed lag model; R (NDVI with 5 months lag) = 0.49, *p* < 0.001	Rodent; GDP and the urbanization rate; climate
		Raohe and Mishan County in Heilongjiang, Chang’an District, and Hu County in Shaanxi (county) [58]; 2002–2012	Seasonal autoregressive integrated moving average model with exogenous variables; NDVI with 1-month lag is significantly associated with HFRS cases in Raohe, NDVI with 2-month lag is significantly associated with HFRS cases in Chang’an	Meteorological factors
		Changsha (total area) [29]; 2004.01–2011.12	Autoregressive integrated moving average model; positive correlation between NDVI and HFRS host density with 3 months lag	Meteorological factors; rodents; TVDI
		Changsha (total area) [59]; 2005–2010	Cross-correlation analysis; NDVI values for rice paddies, orchards, forest land, and residential areas were significantly correlated with the monthly notified number of HFRS cases with a lag time of 1–6 months	Meteorological factors; climate factor
		Dayangshu District (the total area) [60]; 2001–2005	Linear regression analysis; R = 0.67 between HFRS cases in farmland and 3 months backward NDVI, *p* < 0.001	None
	NDVI (−)	Changsha (nearly 1 km grid) [23]; 2005–2009	Ecological niche models; NDVI value in areas predicted present is lower than in areas predicted absent	Elevation; temperature; precipitation; land use; rodent density
		Hunan Province (1 km grid) [43]; 2010–2019	Binary logistic regression model; B = 0.563, *p* < 0.01, 1st influencing factor, negatively correlated	Soil; land use; altitude; population (3rd); precipitation; GDP (2nd)
		Hebei Province (city) [61]; 1999–2011	Correlation analysis; R = −0.463, *p* < 0.05	Rodents; meteorological factors
Land use—Husbandry	Deer husbandry (+)	Changchun City (total area) [62]; 1998–2012	Poisson regression analysis; Shuangyang County, HFRS incidence increased by 70.7% as the deer density increased by 10 head per km^2^ (*p* < 0.001); combined other nine counties, HFRS incidence increased by 90.4% as the deer density increased by 10 head per km^2^ (*p* < 0.001)	Climate factors
	Pig density (+)	Shaanxi Province (county) [25]; 2005–2016	Multivariate boosted regression; relative contribution = 9.86%; a trend of firstly decreasing and then increasing	Meteorological factors; elevation; GDP; land use (1st), population density (3rd); cattle density; goat density; selenium
Vaccination	Expanded program of immunization (−)	China (total area) [34]; 2005–2010	The proportion of HFRS cases among EPI-targeted age group decreased from 86.9% in 2005 to 81.9% in 2010; unvaccinated group increased from 13.1% to 18.1%; the differences were significant	None
	Expanded program of immunization (−)	Yichun City (total city) [35]; 2005–2013	HFRS incidence of EPI-targeted population remained stable while incidence of population in non-EPI-targeted regions and non-EPI-targeted population in targeted region presented increasing tendency	Rodents
	Vaccination (−)	Hu County (total county) [22]; 1984–2014	Significant decrease in the number of susceptible individuals observed after the mass vaccination	Rainfall; resource availability; rodents
	Vaccination (−)	Hu County (total county) [63]; 1971–2011	Cross-correlation analysis; negative correlation between HFRS incidence and vaccination compliance with lags of 1 and 2 years (R = −0.51 and −0.55)	None

+, positive correlation with HFRS epidemic; −, negative correlation with HFRS epidemic; +, −, firstly positively correlated and then negatively correlated; risk area, with high HFRS epidemic risk in a specific value area. Gross domestic product (GDP); normalized difference vegetation index (NDVI) reflects the comprehensive situation of natural environment and human social and economic activities. 1st, the contribution rate of the influencing factor rank as the first in the model; 2nd, the contribution rate of the influencing factor rank as the second in the model; 3rd, the contribution rate of the influencing factor rank as the third in the model.

## Data Availability

Not applicable.

## Supplementary Materials

The following supporting information can be downloaded at https://www.mdpi.com/article/10.3390/ijerph20043328/s1, Table S1: quality assessment table.
